# Insights into countries’ exposure and vulnerability to food trade shocks from network-based simulations

**DOI:** 10.1038/s41598-022-08419-2

**Published:** 2022-03-17

**Authors:** Marco Grassia, Giuseppe Mangioni, Stefano Schiavo, Silvio Traverso

**Affiliations:** 1 Department of Electric, Electronic and Computer Engineering, University of Catania, Catania, Italy; 2 School of International Studies, University of Trento, Trento, Italy; 3 Department of Economics and Management, University of Trento, Trento, Italy; 4 OFCE-DRIC, Paris, France; 5 Department of Economics, University of Genoa, Genoa, Italy

**Keywords:** Environmental economics, Environmental sciences, Environmental social sciences, Applied physics, Statistical physics, thermodynamics and nonlinear dynamics

## Abstract

In the context of a global food system, the dynamics associated to international food trade have become key determinants of food security. In this paper, we resort to a diffusion model to simulate how shocks to domestic food production propagate through the international food trade network and study the relationship between trade openness and vulnerability. The results of our simulations suggest that low-income and food insecure countries tend to be the more exposed to external shocks and, at the same time, they are usually not in a position to take full advantage of international food trade when it comes to shield themselves from shocks to domestic production. We also study and discuss how nodes characteristics are associated with the propagation dynamics and with countries’ vulnerability, finding that simple centrality measures can significantly predict the magnitude of the shock experienced by individual countries.

## Introduction

Despite the substantial progress achieved over the last 30 years, food insecurity still affects about two billion people worldwide and the prevalence of undernourishment has been slowly on the rise since 2014. According to FAO and other international agencies, this alarming trend is likely due to a mix of factors, but mainly driven by the interaction between a surge in the number of conflicts and increasing climate-related shocks^1,2^.

With around one-quarter of agricultural production moving across international borders^3^ and the emergence of a truly global food system^4^, international trade has become a key determinant of food security and plays a crucial role in the transmission of shocks, both natural and man-made^5,6^. On the one hand, international flows allow countries to decouple food consumption from local production, to exploit their comparative advantages and to make a more efficient use of their natural resource endowment^3,7–11^. In addition, food trade can contribute to the mitigation of the risks associated with idiosyncratic shocks to domestic food supply^12–16^. On the other hand, interconnected markets can facilitate the transmission of shocks and their diffusion, thus increasing the vulnerability of countries to disruptions originating from abroad^17–19^. The role of trade is particularly sensitive when it comes to agricultural goods, as there is a long-standing debate on the merits of international integration versus some degree of ‘food sovereignity’^20–23^, and export restrictions are a common, albeit often ineffective, tool to address shocks to domestic supply^24^.

In this paper, we use a network model of shock diffusion to investigate this double role of trade in mitigating vs. propagating local shocks to agricultural production. In particular, we use data on bilateral trade flows to build the international food trade network and then run a series of simulations to explore how the characteristics of individual countries as well as the topology of the network are associated with the spreading dynamics. More precisely, by simulating a series of shocks to domestic and foreign food production, we study whether and to what extent a country’s position in the food trade network can predict its exposure to external shocks as well as its ability to mitigate the effects of a fall in domestic food production by sharing the burden with the other countries in the network. Finally, we identify the level of reserves capable of guaranteeing an adequate level protection from most of the shocks we simulated.

As climate change increases the likelihood of extreme weather events that can adversely affect agricultural production^5,25^, understanding the role of international trade as a mitigating factor or, on the contrary, as a source of local and global vulnerability is of paramount importance. The 2021 *World Trade Report* by the WTO recognizes that trade can contribute to spread shocks and country characteristics, such as the level of trade diversification, are important determinants of the role played by international flows^15^.

In this paper we move one step further and look at the role of a country’s location within the network as a further determinant of exposure to external shocks. We postulate that countries with the same number of trade partners and a similar degree of openness can be more or less vulnerable to a distant shock, or contribute differently to spread it, depending on their topological features^26^. We describe international food trade as a directed graph in which the edges are weighted according to the total amount calories embedded in bilateral trade flows. Overall, the results of the analysis contribute to the strands of literature that, from different perspectives, study the resilience of food systems^27–35^.

Increased globalization is found to decrease the vulnerability of the food system^27^, although in some regions of the world importing countries have experienced a higher volatility of supply^33^, and growing reliance on a small number of exporters can lead to potential risks^34^. Food system resilience can be measured along different dimensions^30^, namely socio-economic access to food, biophysical capacity and production diversity, and no country features high or low values in all three domains. This multifaceted definition of resilience partly explains the different results in the literature. Reserves are found to play an important role to absorb shocks to the production and global availability of cereals^28,36^. The possibility that sudden decrease in agricultural production can lead to cascades propagating through the trade network has been addressed both looking at a single source of the shock (e.g. the US^32^) or looking across several countries^19^. The spreading dynamics crucially depend on the topology of the network, in particular the presence of densely connected communities^31^. Finally, the structure of the global food system and its dynamic evolution can be modelled as a preferential attachment network: the analysis suggests that while the system displays low resilience, over time it becomes less vulnerable to attacks^29^.

We extend on the existing literature by looking at all food items rather than concentrating on a small set of staple foods, simulating the impact of shocks originating (in turn) from all countries, and by considering that a countries’ income will influence the distribution of shocks across trading partners. Anticipating the results, our simulations indicate that, given the topology of the international food trade network and under the assumptions of our diffusion model, African and Middle-East countries, together with some clusters of countries in Latin America and East Asia, tend to be the most vulnerable to international food trade shocks. In fact, in the case of external shocks (that is, shocks originating in foreign countries and ‘imported’ via international trade), this group of countries pays the highest price in terms of per capita reduction in food availability. On the other hand, even though international food trade represents a valuable channel to hedge against idiosyncratic shocks, African and Asian countries seem to be less equipped to take full advantage of this mechanism.

Finally, by analysing how node characteristics relate with the simulation results, we find that standard centrality measures, such as node degree and node strength, correlate significantly with the severity of caloric deficits. Higher order measures, such as hubness, betweenness and PageRank, are also significant, but to a lesser extent.

## Results

### Exposure to food trade shocks

In order to assess countries’ exposure to food trade shocks we simulate a 30% drop in domestic food production for each country at a time, and study how the shock propagates through the network. At the end of each simulation, some countries in the network (one, at least) will register a demand deficit, that we measure in terms of daily calories per capita (kcals/person/day). Hence, by calculating the average deficit across all the simulations, we obtain an indicator of countries’ overall exposure to food trade shocks.

**Figure 1:**
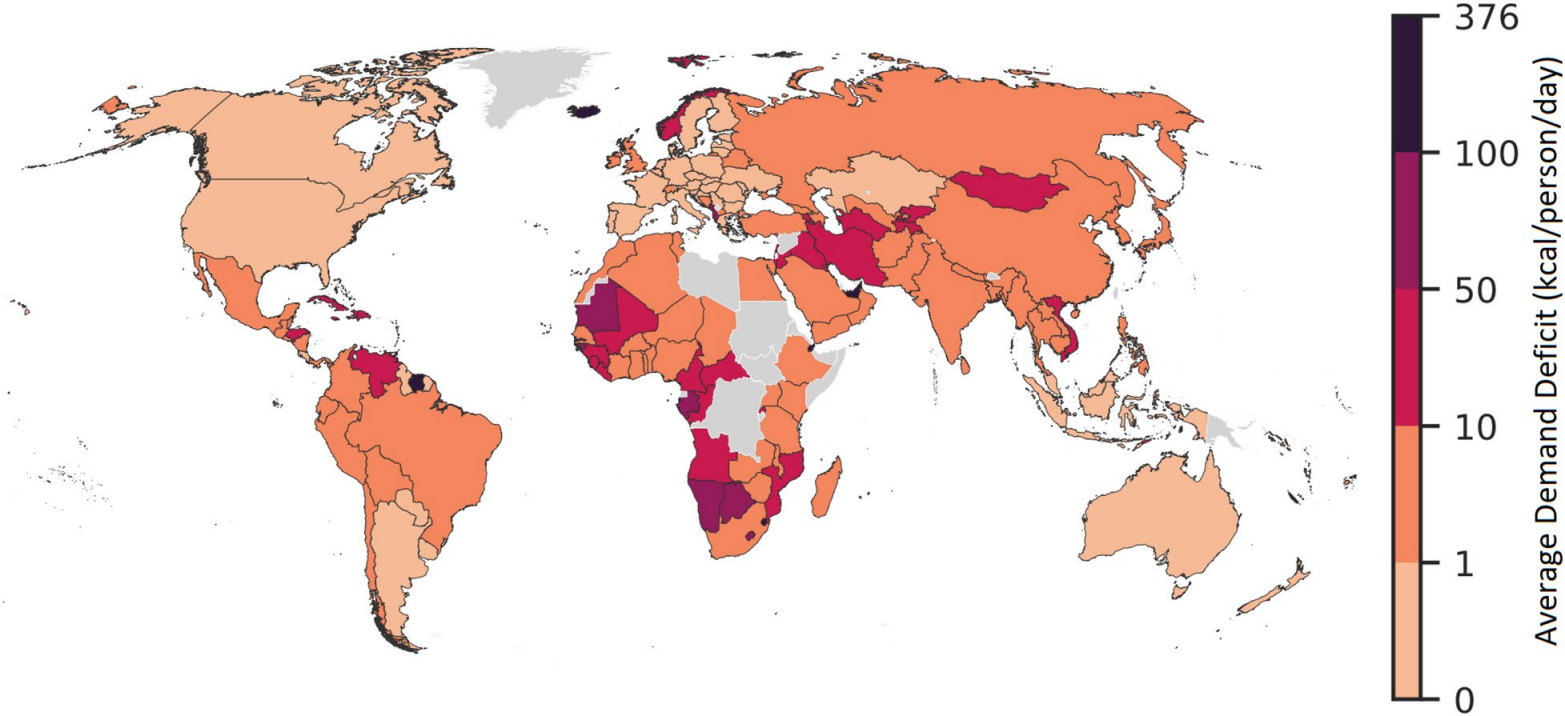
Exposure to external shocks: average demand deficit. The map reports the final demand deficit (in terms of kcals/person/day) averaged over a series of *N* simulations that reproduce, one at the time and for all the countries in the network, the propagation of shock associated to a 30% drop of domestic food production. The diffusion of the shock is simulated setting $\alpha =1$, but similar results can be obtained imposing $\alpha =0.5$ and $\alpha =0$. The map is generated by using GeoPandas^37^ with Natural Earth data v4.1.

As shown in Fig. 1, for most of the countries in the network, the expected demand deficit associated to an idiosyncratic shock hitting a random node is close to zero or below 10 kilocalories per person per day. It is also apparent, however, that most of the exposed countries are concentrated in Africa, in the Middle East, in East Asia and in Latin America and the Caribbean. The highest demand deficits are concentrated among a small number of countries—many of which heavily rely on food import and are characterized by low level of income and food security—that struggle to pass on the shock to their trade partners.

**Figure 2:**
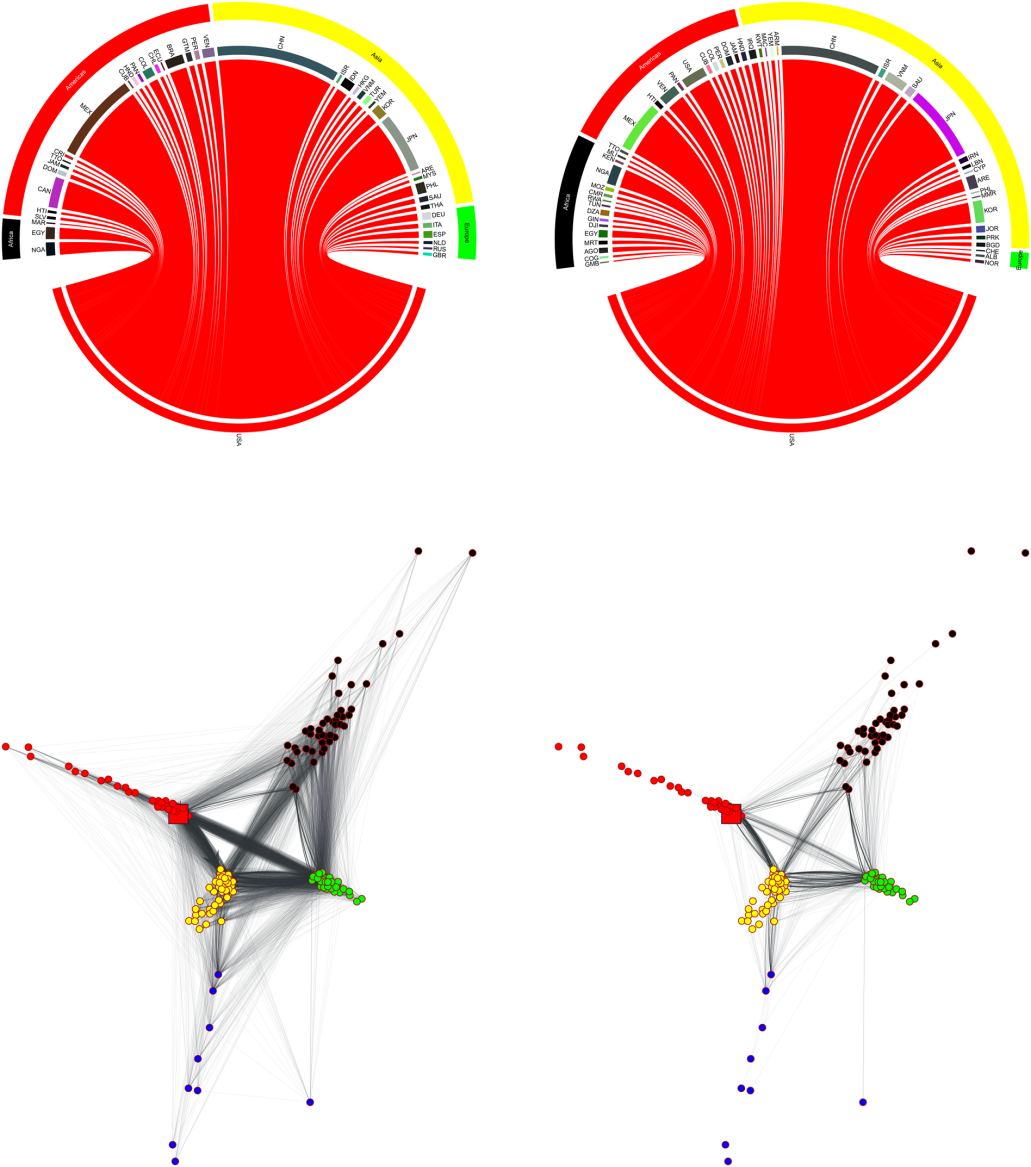
Diffusion of shock hitting the US: first (left panel) and last (right panel) step of the simulation. The two chord diagrams show, in absolute terms (kcals), how a simulated shock associated to a 30% reduction in US domestic food production propagates to the other countries of the network. In particular, the left panel reports the countries affected in the first step of the simulation, while the right panel reports the final distribution of the shock (i.e., at the end of the simulation). The diffusion of the shock is simulated setting $\alpha =1$, but similar results can be obtained imposing $\alpha =0.5$ and $\alpha =0$. Below the chord diagrams, the corresponding networks are shown. The color of the nodes represents the Country’s continent, and the mapping is the same as in the chords diagrams. The node marked by a square represents the US.

To illustrate the diffusion process with a specific example, Fig. 2 presents the case of a 30% decline in US food production. In the first step of the simulation (left panel), the shock is transmitted only to direct trade partners, which are mostly in Asia and in the Americas. After that, however, the shock reaches also countries that do not directly trade with the spreader and, by the end of the simulation (i.e., when no country can further cut its exports in order to reduce its demand deficit), the burden has partially shifted towards (mostly poor) food-importers in Africa and Asia (right panel of Fig. 2). For example, Canada and Germany are able to pass the shock on to their trade partners and do not experience demand deficits, while countries such as Algeria, Angola, Mauritania and Mozambique, despite not directly importing food from the US, end up absorbing a significant portion of the shock. Interestingly, because of the complex diffusion dynamics, at the end of the simulation part of the shock returns to (and it is absorbed by) the spreader.

Networks shown in the lower panel in Fig.  2 can also help in visualizing the effect of the shock originated in the United States. What emerges is a significant reduction in the number and the strength of bilateral links by the end of the simulation. This translates into a number of isolated nodes, corresponding to food-importers that are mostly located in Africa and Asia. Such a modification of the network is also evident by looking at the node degree and strength distributions, reported in Fig.  5. It is interesting to note that, while the degree distribution is heavily modified, only minor changes can be observed in the strength distribution. From a modeling point of view, this result stems from Eqs. (8) and (9) of our shock propagation model. In fact, they express that the fraction of demand deficit absorbed by an importer-country is inversely proportional to its GDP. The impact of this policy is particularly strong on countries with low GDP and weak links that become rapidly isolated. In other words, small and poor countries that import a lot of food while exporting little bear the brunt of any shocks since they will be heavily affected by export reductions imposed by their partners while at the same time not being able to transfer a relevant share of the shock to other countries. When countries that are already peripheral to the world trade system are hit, reductions in trade flows can result in link removal, with a number of countries becoming isolated. Because links that are removed are typically weak to start with, the impact on the strength distribution is more limited compared to the degree distribution. Qualitatively similar patterns are observed across all countries, with node-specific features such as the position within the network and the amount of food export determining a country’s ability to pass on the shock to trade partners and third countries.

### Hedging against domestic shocks

When a country is integrated into international food markets, it is exposed to the risks of external shocks. At the same time, however, international trade ensures that it can rely on food import to compensate unexpected declines in its domestic production. In other words, food trade can hedge countries against the risk of idiosyncratic shocks to agricultural output that may happen, for example, in the wake of adverse weather conditions or natural disasters.

**Figure 3:**
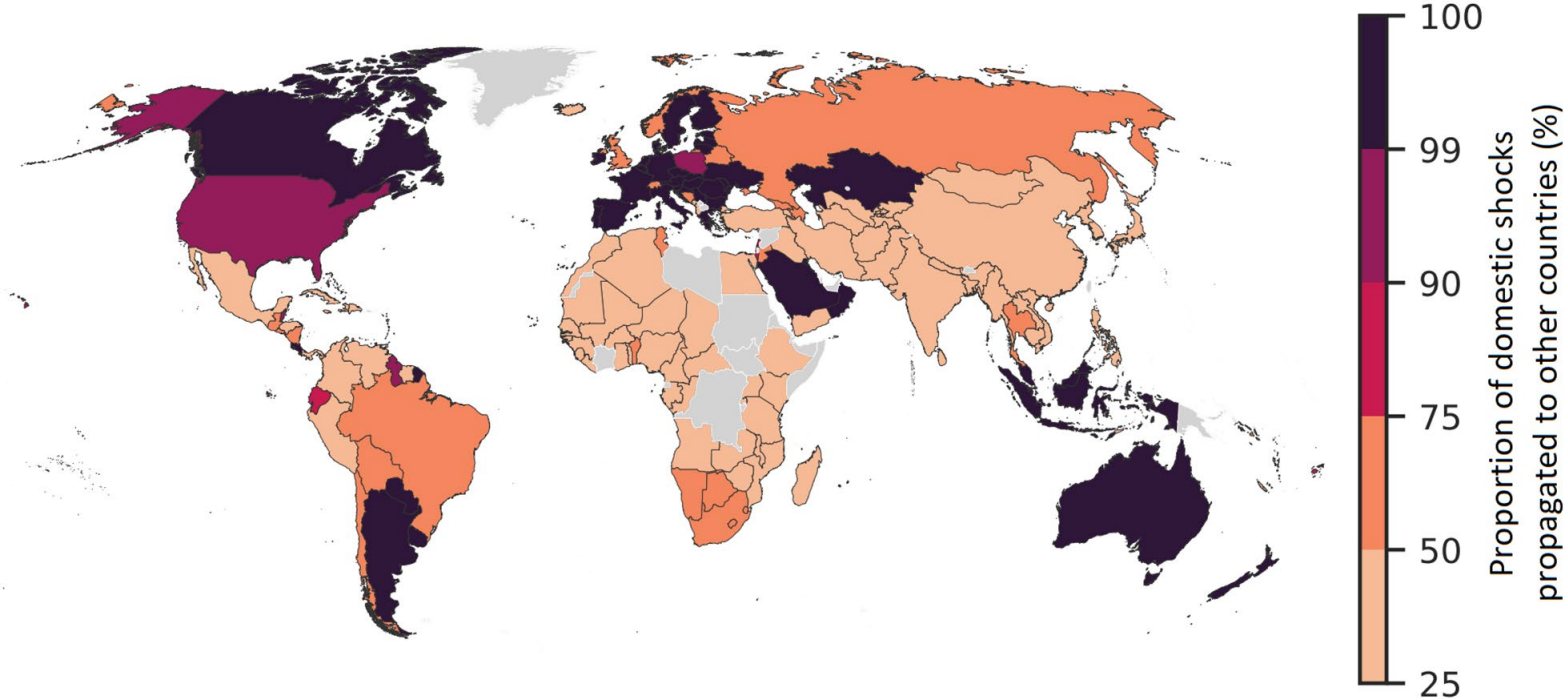
Hedging against domestic shocks: proportion of domestic shocks propagated to other countries. The map reports the proportion of the domestic shock (a 30% drop in domestic food production) that each country manages to pass on to the rest of the trade network. The diffusion of the shock is simulated setting $\alpha =1$, but similar results can be obtained imposing $\alpha =0.5$ and $\alpha =0$. The map is generated by using GeoPandas^37^ with Natural Earth data v4.1.

Not all the countries, however, have the same capacity to take advantage of international trade. Indeed, as shown in Fig. 3, while some countries are able to shift a large share of their domestic shocks to the rest of the network, others struggle to do so. On the one hand, most European countries, together with those in North America and in the Pacific region, are able to resort to trade to deflect shocks without serious consequences on domestic food availability. For example, even in the case of a 30% fall in domestic production, the US would manage to shift more than 90% of the burden on the rest of the network, ending up absorbing only a small fraction of the original shock (see also Fig. 2). On the other hand, according to our simulations, several other countries are not able to take full advantage of the trade channel to ease the pressure of a fall in domestic food supply, ending up with a large reduction in the amount of available calories. As in the case of exposure to external shocks, these countries are concentrated in Africa, in the Middle East and in the continental part of South-East Asia, that are the regions where most of the food insecure households live. Indeed, most of these countries barely manage to share 25% of the burden of the food crisis with the rest of the network.

### Food shocks and emergency reserves

The adverse effects of food shocks can be greatly mitigated when countries can rely on emergency food reserves. Indeed, adequate buffer stocks can smooth out unexpected reductions in food supply. Our simulations allow to identify the amount of reserves to stock to insure against most of the shocks. To do so, for each country we take the 95th percentile of the demand deficits stemming from the entire set of simulations across the different values of $\alpha \in (0, 0.5, 1)$, and use it as a measure of the reserve stocks needed to fend off all but the most severe outcomes of the simulations.

Table 1 reports a list of the 40 countries (with a population of at least one million) with the highest demand deficits (in terms of kcal/person/day) corresponding to the 95th percentile of the simulations. Similarly to what has emerged from Figs. 1 and 3, poor countries are more likely to endure the most severe deficits. Moreover there is some geographic clustering in the results: out of the 40 countries included in Table 1, 15 are located in West and South Africa, 7 in the Middle east, 7 in Latin America and the Caribbean.

The simulations suggest that, in order to able to deal with 95% of the shocks, the countries listed in Table 1 need to stock substantial amount of food reserves. For example, a country like Cyprus should store an amount of food equivalent to about thousand kilocalories per person per day, corresponding to about one fourth of the country’s domestic food supply. While most of the countries on the top the list are relatively small, the list also includes a number of large countries such as Vietnam, Congo and Iraq. On the one hand, this problem could be best addressed by constituting international emergency funds to help the countries facing the worst shocks. On the other hand, coordination problems are often exacerbated in times of crisis and even if pooling food reserves represents the first best solution, it might be difficult to achieve in practice^38^.

**Table 1:** Demand deficits in worst-case scenarios.

	Demand deficit	As % of total food supply (%)		Demand deficit	As % of total food supply (%)
Cyprus	1069	25.2	Guinea	145	5.3
United Arab Emirates	747	17.3	Kyrgyzstan	142	5.2
Trinidad and Tobago	585	19.4	Turkmenistan	135	4.9
Albania	545	15.6	North Macedonia	126	4.2
Jordan	478	18.1	Cuba	116	3.4
Mauritania	464	15.8	Haiti	115	5.5
Gabon	428	16.4	Angola	114	5.6
Kuwait	387	11.6	Rwanda	99	4.1
Gambia	302	12.2	Dominican Republic	95	3.5
Guinea-Bissau	296	12.5	Sierra Leone	92	4.3
Congo	287	13.5	Jamaica	92	3.4
Lebanon	281	11.3	Mali	89	3.3
Armenia	229	7.6	Mozambique	82	3.5
Eswatini	191	7.1	Viet Nam	72	2.6
Mongolia	181	7.3	Honduras	71	2.9
Norway	160	4.6	Lesotho	68	2.6
Timor-Leste	156	7.5	Cameroon	68	2.5
Israel	156	4.3	Tajikistan	65	2.9
Iraq	150	5.8	Venezuela	58	2.1
Liberia	148	6.6	Yemen	54	2.5

The table reports the countries which, in a worst-case scenario, experience the highest demand deficit in terms of kcal/person/day. The overall demand deficit is also reported as a percentage of total domestic food supply. The worst-case scenarios are identified by taking the 95th percentile (reported in terms of kcal/person/day) of the distribution of country-level demand deficits. The distribution of the country-level demand deficits pools the deficits resulting from three sets of simulations based on different values of the $\alpha$ parameter ($\alpha =0, 0.5, 1$). The list includes only countries with a population of at least one million.

### Regression analysis

To get a better sense of the relationship between a country’s position in the network and the outcome of the simulations, we run a series of regressions where the dependent variable is the demand deficit (kcals/person/day) faced by each country *j* when a shock hits country *i*. Considering the large number of zeros in the outcome variable, we employ Tobit regression instead of standard OLS. A detailed description of the econometric methodology is presented in “Methods” section.

Explanatory variables include per capita GDP, absolute shock size, the concentration of import flows, indicators for whether the shock is domestic, or it originates in a partner country, and standard network measures for both countries (the one hit by the original shock and the one for which we measure the demand deficit).

**Figure 4:**
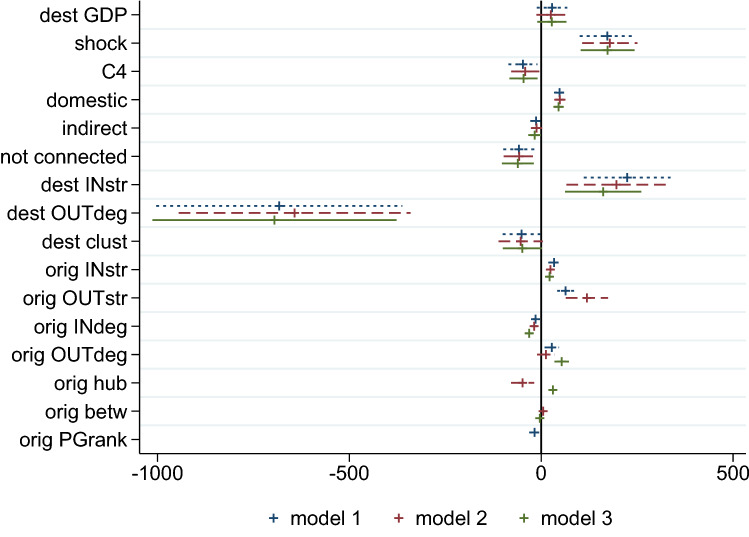
Demand deficit and node characteristics: overview of Tobit regression results. Standardized coefficients from Tobit regression on the determinants of demand deficit. Explanatory variables with non-significant coefficients not reported (full regression results available in Table 5 of “Methods” section).

The main results are summarized in Fig. 4, where we compare three different econometric specifications based on the inclusion of alternative centrality measures. Details of the regression results, including explanatory variables with non-significant coefficients, are presented in Table 5 in “Methods” section.

Predictably, larger shocks tend to be associated with larger demand deficits. When the shock is domestic, a country will—everything else equal—bear a larger fraction of it and thus experience a larger caloric deficit. On the other hand, when the shock originates abroad, we have two indicator variables, one denoting an indirect connection between the origin of the shock and the one experiencing a demand deficit (that is, $distance(i,j) \ge 2$), and the second taking value 1 if there is no direct path linking *i* to *j*. A shock hitting a non-direct partner has, on average, a lower effect on any country. However, the small a barely significant coefficient suggests that in a highly connected system, the difference is minimal and shocks reverberate globally.

The concentration of imports (share coming from the four most important partners, C4), has a negative effect, suggesting that relying on a small number of key suppliers shields countries from external shocks and diversification seems to have little beneficial effects in our simulations.

When shocks originate in a country with a large number of outward links, it has multiple channels to propagate through. As a result, other countries end up with larger demand deficits. On the contrary, many inward links imply that a higher proportion of the shock remains in the originating country.

Of course, the number of import partners is a crude measure of exposure to external shocks, because not all trade flows are equally important. When looking at the total magnitude of kcals that are either imported or exported by countries, we observe that a larger amount of imports is associated with a larger demand deficit, and the same holds when a shock originates from a country that exports a lot. On the other hand, this feature does not play a significant role in shielding countries from the negative effects of a shock (although the estimated coefficient for the out-strength of destination countries takes a negative sign, it is not significant at 5% and thus not reported in Fig. 4).

A high level of clustering is associated, on average, with lower demand deficits. One way to rationalize this result is that when a shock reaches a cluster, there are multiple channels that allow countries to dissipate it; moreover, it is less likely to disproportionately affect a single partner since there are strong interconnections within the cluster. We experiment with different measures of network centrality, namely betweenness, hub score, and PageRank. We do not have a strong prior on the effect of centrality: while shocks originating in more central nodes will probably affect a larger number of countries, the very fact that they travel further implies there is more room to dissipate and have smaller effects on single nodes. On the other hand, more central countries are more likely to be hit by external shocks, but should also have the ability to pass them on to more partners.

We find no significant correlation between any of the centrality measures and the size of the final demand deficit in countries receiving the shock. On the contrary, the position of origin countries in the network does play a role. Hub score and, to a lesser extent, PageRank centrality are negatively correlated with final deficits, whereas the correlation is positive when we look at betweenness centrality. Hubs, which export to several countries, thus appear to distribute the shock across multiple partners and this reduces the toll imposed on each one of them. On the other hand, high betweenness may signal the ability of a country to connect different parts of the network, but not necessarily through many links; as a result, when a shock originates in a central country it may travel to distant countries yet generate, everything else equal, important consequences on trade partners.

The coefficients for origin country’s hub centrality and out-strength have opposite sign in the regression, which may seem counter-intuitive. Yet, the coefficient on hub centrality turns negative only when origin out-strength is included in the regression, while taking a positive value otherwise. A possible interpretation is that while out-strength captures the overall ‘fire power’ of a node, and so its ability to diffuse/transmit the shock, hub centrality also depends on the identity of network partners (in the present context, hubs are countries that export a lot to big importers). If large importers somehow limit the transmission of shocks (because they export less and so have less room to reduce their sales abroad), then shocks hitting hubs will travel a smaller distance in the network. We also note that this result is driven by a few countries with very high centrality (both in terms of hub and out-strength): if we drop observations in the top 10% of hub centrality, the associated coefficient turns positive (yet not significant).

### Vulnerability analysis

In this part of the analysis we concentrate on the vulnerability of countries to external shocks, by taking the vantage points of ‘destination countries’ and pooling across all the simulations. We follow two complementary approaches: first we look at the number of times a country experiences a caloric deficit above a critical threshold; then, we analyze the ranking of each country in terms of demand deficit.

#### Demand deficit above a critical threshold

We have defined two threshold levels for the final demand deficit in each country, equal to either 250 or 500 daily kcals per capita: these represent 1/8 or 1/4 of the recommended daily intake for an adult. We count how many times a given country faces a demand deficit greater or equal to the threshold across all the simulations we run, and use the value as the dependent variable capturing the vulnerability of countries. In choosing the threshold we have to balance the severity of the nutritional deficit against the need to have enough observations for which the dependent variable is nonzero. Because the dependent variable is a count, we cannot use standard OLS but rather revert to a negative binomial regression. Table 2 displays results for the two thresholds. Estimated coefficients are similar both qualitatively and quantitatively, suggesting that the value of the threshold is not crucial to the results.

Per capita GDP is positively correlated with our measure of vulnerability: while counter-intuitive at first, this result is driven by the fact that richer countries are more heavily embedded in international trade and thus more likely to suffer from shocks coming from different sources, and they tend to import more calories. The number of trading partners does not play any role in determining the number of times a country suffers a large demand deficit, nor does the total amount of calories that are imported. On the contrary, node out-strength (that is, total exports) reduces vulnerability as it provides countries with more room to pass on a shock to trade partners. A similar negative correlation is found for the concentration of imports (measured in terms of C4) and clustering. We interpret these findings as a signal that relying on a small number of source countries protects countries from shocks originating in other parts of the network and is consistent with results discussed in "Regression analysis". Because in our exercise shocks originate in turn from all possible countries, being exposed to only a few of them turns out to reduce the number of instances where the final demand deficit is large. Similarly, clustering is associated with tight links among trading partners indicating both the possibility to pass on the shock to other countries and a more widespread diffusion of disturbances, that are less likely to generate large disruptions. Finally, among the different measures of centrality we include in the analysis (betweenness, hub score and PageRank) only betweenness is positively correlated with the number of times a country experiences a large caloric deficit. In this case the results are more significant when using 500 kcals/person/day as a threshold, the only significant difference between the two parts of Table 2.

**Table 2:** Vulnerability analysis: negative binominal regression.

Threshold	250 kcals/person/day					500 kcals/person/day				
	(1)	(2)	(3)	(4)	(5)	(6)	(7)	(8)	(9)	(10)
log pcGDP	0.434***	0.426***	0.440***	0.420***	0.399***	0.497***	0.470***	0.481***	0.478***	0.452***
	(0.118)	(0.117)	(0.121)	(0.120)	(0.117)	(0.134)	(0.126)	(0.135)	(0.137)	(0.128)
INdegree	− 0.016	− 0.019	− 0.018	− 0.021*	− 0.022*	− 0.023	− 0.027*	− 0.024	− 0.029*	− 0.031**
	(0.013)	(0.012)	(0.013)	(0.012)	(0.012)	(0.016)	(0.016)	(0.016)	(0.016)	(0.016)
OUTdegree	− 0.002	− 0.005	− 0.005	− 0.006	− 0.007	0.004	− 0.000	0.002	− 0.000	− 0.000
	(0.005)	(0.005)	(0.005)	(0.005)	(0.005)	(0.006)	(0.007)	(0.007)	(0.007)	(0.007)
log INstrength	− 0.161	0.127	0.129	0.130	0.132	− 0.179	0.144	0.095	0.146	0.126
	(0.124)	(0.154)	(0.160)	(0.149)	(0.152)	(0.151)	(0.188)	(0.199)	(0.181)	(0.187)
log OUTstrength	− 0.356***	− 0.413***	− 0.379***	− 0.379***	− 0.420***	− 0.422***	− 0.544***	− 0.456***	− 0.462***	− 0.545***
	(0.073)	(0.072)	(0.070)	(0.072)	(0.073)	(0.090)	(0.089)	(0.088)	(0.091)	(0.093)
Imports C4	− 2.922***	− 3.624***	− 3.519***	− 3.490***	− 3.619***	− 2.982**	− 3.785***	− 3.355***	− 3.604***	− 3.697***
	(0.949)	(1.036)	(1.027)	(0.970)	(1.005)	(1.254)	(1.315)	(1.291)	(1.229)	(1.232)
Clustering		− 2.475**	− 2.788***	− 3.151***	− 2.899***		− 2.424*	− 2.700**	− 3.307***	− 2.597*
		(1.007)	(0.959)	(0.990)	(1.088)		(1.337)	(1.271)	(1.215)	(1.451)
Log betwenness		0.063			0.074*		0.136**			0.141***
		(0.043)			(0.041)		(0.053)			(0.048)
Hub score			0.333		0.664			− 16.506		− 9.460
			(3.057)		(2.129)			(20.967)		(17.291)
PageRank				0.041	0.050				0.039	0.055
				(0.032)	(0.033)				(0.054)	(0.059)
Observations	516	516	516	516	516	516	516	516	516	516

The table reports the results on the negative binomial regressions in which the dependent variable is the number of times a country suffers demand deficits higher than 250 and 500 kcals/person/day. Each simulation represents a shock associated to a 30% drop of domestic food production starting from a specific country and diffusing through the network with a given value of the parameter $\alpha$. We have 172 countries and set $\alpha \in (0, 0.5, 1)$ for a total of 516 simulations. Dummies for $\alpha \in (0, 0.5, 1)$ not shown. Clustering and PageRank measures have been multiplied by 1,000. Robust standard errors in parentheses. Significance level: ***$p<0.01$, **$p<0.05$, *$p<0.1$.

#### Ranking analysis

To further investigate the vulnerability of countries, we rank them based on the size of the final demand deficit they face in each simulation, with the smallest deficit taking value 1. We then sum across all the simulations and take logs to obtain a continuous measure of country vulnerability, which we regress on the same set of correlates used in the previous section.

Results in Table 3 are qualitatively similar to those obtained with the negative binomial regression, although the significance of the specific variables is much lower now. Out-strength confirms its role as an escape valve, while hub-score centrality is associated with more vulnerability.

**Table 3:** Vulnerability analysis: OLS regression of ranking.

	(1)	(2)	(3)	(4)	(5)
Log pcGDP	0.019	0.017	0.031	0.018	0.016
	(0.063)	(0.064)	(0.063)	(0.064)	(0.065)
INdegree	0.004	0.003	0.000	0.002	− 0.000
	(0.005)	(0.005)	(0.005)	(0.005)	(0.005)
OUTdegree	0.003	0.004	0.003	0.003	0.003
	(0.002)	(0.002)	(0.002)	(0.002)	(0.002)
Log INstrength	0.061	0.014	0.077	0.018	0.069
	(0.048)	(0.054)	(0.056)	(0.053)	(0.057)
Log OUTstrength	− 0.574***	− 0.582***	− 0.577***	− 0.570***	− 0.583***
	(0.026)	(0.030)	(0.026)	(0.027)	(0.029)
Imports C4	− 0.511	− 0.435	− 0.789*	− 0.468	− 0.754*
	(0.384)	(0.391)	(0.404)	(0.381)	(0.407)
Clustering		0.490	0.008	0.326	− 0.002
		(0.357)	(0.330)	(0.343)	(0.357)
Log betwenness		0.013			0.014
		(0.022)			(0.021)
Hub score			1.839***		1.826**
			(0.647)		(0.738)
PageRank				0.009	0.009
				(0.010)	(0.009)
Observations	516	516	516	516	516
R-squared	0.994	0.994	0.994	0.994	0.994
F stat.	5706	5116	4761	4695	4592

The table reports the results on the OLS regressions in which the dependent variable is countries’ ranking in terms of vulnerability to external shocks (higher values in the ranking are associated with higher vulnerability). The ranking is based on the results of a set of 3*N* simulations that reproduce, one at the time and for all the countries in the network, the propagation of shock associated to a 30% drop of domestic food production and a value of $\alpha$ equal to 0, 0.5, and 1. Dummies for $\alpha \in (0, 0.5, 1)$ not shown. Clustering and PageRank measures have been multiplied by 1,000. Robust standard errors in parentheses. Significance level: *** $p<0.01$, ** $p<0.05$, * $p<0.1$.

## Discussion

The paper examines the role of international trade in food as a vehicle for the diffusion of shocks originating in foreign countries and as a channel to diversify risks and share the burden of domestic shocks with other countries. To do so, we portray international food trade as a directed network of calories and simulate the propagation of the shock by refining the model developed by Burkholz and Schweitzer^19^.

Our simulations indicate that, in a globalized food system, shocks can easily travel a long way across the trade network, so that they end up affecting countries which are far away from the original spreader. The results of the analysis suggest that low-income and food insecure countries tend to be more exposed to external food trade shocks. Indeed, on average, high income countries turn out to be better equipped to deflect shocks originating from abroad and therefore, when a shock hits the network, it is more likely that it will be absorbed by countries in Africa, in the Middle East or in Latin America. At the same time, even if the participation to international food trade always provide some level of insurance against shocks to domestic food supply, the degree of protection exhibits substantial regional variation. In fact, while most of the countries in Europe, North America and Oceania are able to take advantage of trade to divert the negative effect of domestic shocks, other countries face worse hedging opportunities. In theory, by establishing food reserves, every country could shield itself against negative food shocks. Individual countries, however, may not be able to stock enough food to withstand a worst-case scenario. Even though the constitution of international reserves would be a cost-effective solution to ensure all the participants to the food trade network against negative food shocks, it might not be feasible because of coordination problems, which are often exacerbated by food crises.

We also analyse and discuss in detail how node characteristics are associated with countries’ vulnerability and average exposure. Among other results, we find that while higher-order network measures such as clustering tend to dampen the effect of trade shocks, the number of trading partners does not seem to play a relevant role in determining countries’ vulnerability. In this respect, we note that the lack of a beneficial effects from trade diversification may be partly due to our analytical setting, which assigns the same probability to shocks originating from each country. Weighting the likelihood of the shocks using information on past reductions in domestic output or on the probability of adverse weather conditions^39^ may thus represent an interesting extension to the model and an avenue for further research.

## Methods

### Data and network structure

The analysis is based on a set of simulations of the diffusion dynamics of food shocks through a weighted and directed network which is calibrated on the basis of actual international food trade data. More specifically, the network is built to resemble the structure of the international trade of food products, with each node representing a country and the edges representing the bilateral trade flows. The weight of the edge from the generic origin country *i* to the generic destination country *j* is given by the total amount of calories embedded in the food products exported from the former to the latter. The total number of nodes is 172 and no major country is excluded. Data on trade flows refer to 2013, the latest year for which the required information was available at the time of the analysis (September 2019). Given the stability of trade networks^40^, the specific year used for calibration of the diffusion model is not crucial for the results.

In formal terms, we run the simulations on a weighted directed graph $G = (V, E, W)$, where $V=\{c_i: i \in \{1, \ldots , N\} \}$ is a set of nodes ($N=172$), $E = \{(c_i, c_j): i,j \in \{1, \ldots ,N\} \}$ is a set of directed edges between pairs of nodes, and $W = \{W_{c_ic_j}: i,j \in \{1, \ldots ,N\} \}$ is the set of the weights associated with the edges (i.e., the kcals embedded in the export of food from country $c_i$ to country $c_j$).

In order to calculate the amount of calories embedded in food trade flows, we follow the approach of Traverso and Schiavo^10^ and match product-level bilateral trade flows with entries from the United States Department of Agriculture’s (USDA) Food Composition Databases. Absent country-specific conversion factors, this approach exploits the size of the US market and quality of the data to determine the caloric content of arount 380 food products^10^. Specifically, we retrieve product-level bilateral food trade data from the Food and Agriculture Organization’s (FAO) trade matrices. Data on fisheries and seafood, not included in the original FAO dataset, are taken from the Centre d’Etudes Prospectives et d’Informations Internationales’s (CEPII) BACI database^41^.

Socioeconomic attributes of the countries, such as total population and gross domestic product (GDP), come from the FAO statistic databases. In addition, we estimate domestic food production by adjusting FAO Food Balance Sheets’ (FBS) data on domestic food supply for countries’ net international position. More specifically, for each country we compute the net food trade balance (in kcals) and we add it to the aggregated kcals supply calculated using FAO data.

### Shock diffusion model

We model the diffusion of shocks building on the setup of Burkholz and Schweitzer^19^. To formally introduce the model, we first define the equilibrium domestic demand (i.e., before the shock) of the generic county $c_i$ as:

1 $$\begin{aligned} dem_{c_i}(t) = prod_{c_i}(t) + imp_{c_i}(t) - exp_{c_i}(t) \end{aligned}$$

in which $prod_{c_i}$, $exp_{c_i}$, and $imp_{c_i}$ indicate domestic production, export and import respectively, expressed in kcals.

Note that in network terms

2 $$\begin{aligned}&exp_{c_i}(t) = \sum _{j = 1}^N W_{c_ic_j}(t) \end{aligned}$$

3 $$\begin{aligned}&imp_{c_i}(t) = \sum _{j = 1}^N W_{c_jc_i}(t). \end{aligned}$$

By using Eq. (1) we can compute the initial demand of each country at time $t=0$.

If a country $c_s$ is shocked at $t=1$ by a $shock_{c_s}$, a demand deficit $dd_{c_s}(t = 1) = shock_{c_s}$ will result. Country $c_s$ can compensate this shock by reducing its export (thus assuming that domestic demand is served first) and/or by increasing its import to compensate for lower domestic production. Formally:

4 $$\begin{aligned} exp_{c_s}(t=1)= & {} exp_{c_s}(t=0) - \alpha \cdot shock_{c_s} \end{aligned}$$

5 $$\begin{aligned} imp_{c_s}(t=1)= & {} imp_{c_s}(t=0) + (1-\alpha )\cdot shock_{c_s} \end{aligned}$$

where $\alpha \in [0,1]$ is a parameter used to distribute the shock between import and export flows. The value $\alpha = 0$ means that the shock is entirely compensated by increasing imports, while a value of $\alpha = 1$ means the shock is propagated by a reduction of exports. Other values of $\alpha$ indicate how shock is split between imports and exports. For example, a value of $\alpha = 0.2$ means that the $20\%$ of shocks is compensated by reducing exports while the reamining $80\%$ is compensated trough an increase of imports.

Shock compensation by country $c_s$ induces a cascading effect on those countries that import/export from/to $c_s$. In fact, after the initial step, the shock propagates in the network producing a demand deficit in a generic country $c_i$ at time step *t* given by:

6 $$\begin{aligned} dd_{c_i}(t) = dem_{c_i}(t=0) - prod_{c_i}(t) - imp_{c_i}(t) + exp_{c_i}(t). \end{aligned}$$

To face this demand deficit country $c_i$ tries to reduce its export to:

7 $$\begin{aligned} exp_{c_i}(t+1) = max\{exp_{c_i}(t) - dd_{c_i}(t), \; 0\}. \end{aligned}$$

It is easy to see that if imports and exports do not change, the demand deficit equals zero $dd_{c_i}(t) = 0$.

Variations on exports and/or imports are, in network terms, implemented by changing the weights associated with the edges connecting countries, as detailed in Eqs. (2) and (3).

We assume two different dynamics for adjustment via import and export. More specifically, any increase in imports is distributed across partners in proportion to the kcals supplied to country $c_i$ in the baseline scenario ($t=0$). On the other hand, reduction in export flows are transmitted in a way that is inversely proportional to the GDP of out-neighbors. Formally, if $dd_{c_i}(t) > 0$, the reduction in exports will be distributed among the countries that import from $c_i$ as:

8 $$\begin{aligned} W_{c_ic_j}(t+1) = max \{ W_{c_ic_j}(t) - dd_{c_i}(t)\cdot F_{c_ic_j}(t), 0 \} \end{aligned}$$

where:

9 $$\begin{aligned} F_{c_ic_j}(t) = {\left\{ \begin{array}{ll} \frac{ \sum _{c_h\ne c_j} GDP_{c_h}}{\sum _{c_h} GDP_{c_h}}\cdot \frac{1}{k^{out}_{c_i}(t) -1} &{} \text {if } k^{out}_{c_i}(t) \ne 1\\ 1 &{} \text {otherwise} \\ \end{array}\right. } \end{aligned}$$

In Eq. (9) $c_h$ ranges over those neighbors of $c_i$ for which $W_{c_i,c_h}(t) > 0$, $GDP_{c_h}$ is the GDP of the generic out-neighbor $c_h$, and $k^{out}_{c_i}(t)$ is the out-degree of country $c_i$ at step *t* (i.e., the number of positive outward edges departing from $c_i$). $F_{c_ic_j}(t)$ expresses the fraction of demand deficit of country $c_i$ absorbed by country $c_j$, and it is inversely proportional to the GDP of $c_j$ compared to the GDP of the other out-neighbours of $c_i$ (i.e. countries importing from $c_i$). Importantly, assuming that the reduction in exports will be inversely proportional to economic size of the trade partners (measured in terms of GDP) represents a major departure from the existing literature^19^, that models export reductions which are proportional to the pre-shock export shares. Countries, in fact, face different budget constraints^18^ and poor countries are likely to be disproportionately affected by global shortages^42^. Therefore, even though the model works with quantities, it mimics some some of the dynamics that would be observed in models that incorporate prices. Equations (8) and (9) ensures that as soon as trade between two countries $c_i, c_j$ falls to zero, the importer will no longer absorb any remaining shock emanating from $c_i$.

The diffusion process stops when no country facing a positive demand deficit can further reduce its exports. While we do not make specific assumptions regarding the length of each step of the diffusion mechanism, we assume that the whole propagation process stops in a relatively short period of time, that is between a few months and a year, as done in the existing literature^19^.

### Network topology

Here we provide a topological analysis of the directed network used in our experiments to simulate the shock propagation. The network includes data from 172 countries (nodes), and 10.5 K food export (weighted links, in Million kcals/year). Each country exports food to, on average, $\sim 61$ countries, i.e., $35.6\%$ of all countries, for an average of $\sim 123B$ kcals/year. $61\%$ of the links are reciprocal (i.e., given a link $e_{ij}$ between the countries *i* and *j*, the link $e_{ji}$ also exists in the network). There are 47 Strongly Connected Components (SCCs, i.e., sub-networks such that there is a path between each pair of vertices) and the largest includes 126 countries. On the other hand, there is only one giant Weakly Connected Component (WCC, i.e., sub-network such that there is a path between each pair of vertices, ignoring the directionality of links) that includes all countries. While the directed diameter of the network cannot be computed since the network is not strongly connected, the undirected diameter is two. That is, any two countries are up to two hops from each other if the direction of the exchange is not considered. The average (unweighted) Local Clustering Coefficient (LCC, defined as the fraction of possible triangles that exist through a given node) is 0.81, while the Transitivity (defined as the fraction of all possible triangles) is 0.674. We also analyze the assortativity of node degree and strength. Specifically, we compute the node-level Pearson’s correlation coefficient between the degree and the Average Nearest Neighbor Degree (ANND)^43^, and also the one between the strength and the Average Nearest Neighbor Strength (ANNS)^44^.

10 $$\begin{aligned} \mathrm {ANNS^{\alpha /\beta }_i} = \frac{\sum ^{\mathcal {N}^{\alpha }_i}_{j} s^{\beta }_j}{k^{\alpha }_i} \end{aligned}$$

where $\alpha , \beta \in [\mathrm {in}, \mathrm {out}]$, i.e., we consider the different in-out link directions, and $\mathcal {N}^{\alpha }_i$ is the $\alpha$ neighborhood of *i*. Moreover, we also report the aggregated ANNS quantity where $\alpha = \beta = tot$ and the *in* and *out* neighbors are aggregated. For the ANND case, instead of the strength $s^{\beta }_j$ we consider the degree $d^{\beta }_j$.

The correlation coefficient show a strong aggregated disassortativity, meaning that nodes that import and/or export to a large number of countries (that is, have a high total degree) are neighbors to countries that have just a few links, and viceversa. A similar consideration can be made for the correlation between the degree and the $\mathrm {in/in}$ and $\mathrm {in/out}$ ANNDs, while the $\mathrm {out/in}$ and $\mathrm {out/out}$ are weaker but positive. On the other hand, all the strength assortativity coefficients are negative, meaning that countries that export and/or import great quantities of kcals are, on average, linked to ones with lower exports and/or imports.

A summary of the topology measures is reported in Table 4.

**Table 4:** Topological analysis of the 2013 food trade network.

Metric	Value
#Nodes	172
#Edges	10,528
Average degree	61.209
Density	0.358
Average link weight	122,752.619
Reciprocity	0.610
#Strongly connected components (SCCs)	47
#Weakly connected components (WCCs)	1
Size largest SCC	126
Size largest WCC	172
Average (unweighted) clustering coefficient	0.812
Transitivity	0.674
Directed diameter	N.A.
Average shortest path length	1.108
Undirected diameter	2
Undirected average shortest path length	1.503
Degree—$\mathrm {ANND^{tot/tot}}$ assortativity	− 0.958
Degree—$\mathrm {ANND^{in/in}}$ assortativity	− 0.921
Degree—$\mathrm {ANND^{in/out}}$ assortativity	− 0.944
Degree—$\mathrm {ANND^{out/in}}$ assortativity	0.494
Degree—$\mathrm {ANND^{out/out}}$ assortativity	0.368
Strength—$\mathrm {ANNS^{tot/tot}}$ assortativity	− 0.554
Strength—$\mathrm {ANNS^{in/in}}$ assortativity	− 0.528
Strength—$\mathrm {ANNS^{in/out}}$ assortativity	− 0.512
Strength—$\mathrm {ANNS^{out/in}}$ assortativity	− 0.063
Strength—$\mathrm {ANNS^{out/out}}$ assortativity	− 0.098

### Estimation methods

We use alternative regression strategies to address different questions. To start, we employ a Tobit regression to investigate the network measures that correlate more strongly with the demand deficit associated with each country in the various simulation exercises. In this case, each observation is identified by a country pair, the one hit by the initial shock and the one for which we measure the final demand deficit, plus a value of the parameter $\alpha$, which determines whether propagation occurs only via exports, or also through imports.

11 $$\begin{aligned} dd_{ij}(\alpha ) = \beta _1 GDP_j + \beta _2 shock_i + \beta _3 C4 + \gamma \mathbf {D_{ij}} + \delta _1 \mathbf {N_i} + \delta _2 \mathbf {N_j} + \eta _{\alpha } + \epsilon _{ij} \end{aligned}$$

The dependent variable, expressed in terms of kilocalories per person per day, is the estimated reduction of per capita domestic food supply in country *j* purplethat results from simulating a $30\%$ fall of aggregate food production in country *i* using a specific value of $\alpha$.

The list of explanatory variables includes shock size (in kcals per capita/day relative to country *j* population), per capita GDP of country *j*, the concentration of imports of food items in country *j* (captured by the share of its top 4 supplier countries in its total imports, indicator variables ($D_{ij}$) for whether the shock is domestic, originates in a country that is directly linked to country *j*, or in a country located ad infinite (network) distance from *j*. Moreover, we add a number of network measures for both the origin ($N_i$) and destination ($N_j$) country, namely in- and out-strength, in- and out-degree, hub score, page-rank, betweenness and clustering. Finally, we control for the value of the parameter $\alpha$.

Tobit regression is chosen because it accommodates the large number of zeros observed in the dependent variable, while assuming that zeros and positive values follow the same data generating process, that is, the same factors explain the zeros and the positive outcome.

The second step of the analysis focuses on a count variable: how many time does a country face a demand deficit higher than 250 or 500 kcals per capita/day, which amount to 1/8 or 1/4 of the recommended daily intake for an adult. OLS regression would be inappropriate in this case and we therefore opt for negative binomial regression, which is preferred over Poisson as it is more robust.

In this case the estimating equation reads as follows:

12 $$\begin{aligned} {{ Large }\, dd_{j}(\alpha )}&= \beta _1 GDP_j + \beta _2 INdeg_j + \beta _3 OUTdeg_j + \beta _4 INstr_j + \beta _5 OUTstr_j \\&\qquad +\beta _6 C4_j + \beta _7 clust_j + \beta _8 betw_j + \beta _9 Hub_j + \beta _{10} pgRank_j + \eta _{\alpha } + \epsilon _{j}. \nonumber \end{aligned}$$

Last, our analysis of the determinants of country vulnerability to shocks ranks each country in terms of the demand deficit they face for each simulation, and then uses the sum across all simulations as the dependent variable, with larger numbers associated with larger vulnerability.

In this case, the dependent variable can be treated as a continuous measure, so that standard OLS regression can be adopted. Apart from the change in the dependent variable, the specification is the same as in Eq. (12).

### Additional results

#### Shock size relative to destination countries’ population

 Predictably, larger global shocks tend to be associated with larger demand deficits. More precisely, a one percent increase in the magnitude of the shock (in per capita terms of the destination countries’ population) is linked to a daily demand deficit increase of about 60 kcals per capita.

**Table 5:** Tobit regression: determinants of demand deficits (kcals/person/day).

	(1)	(2)	(3)
Dest GDP	1.487	1.313	1.462
	(1.065)	(1.007)	(1.021)
Shock	58.117***	60.268***	58.346***
	(12.388)	(12.406)	(12.091)
Concentration (C4)	− 298.728**	− 260.371**	− 288.987**
	(120.680)	(117.063)	(116.963)
Domestic	630.774***	639.228***	594.449***
	(96.645)	(97.462)	(89.017)
Indirect	− 27.906*	− 25.253*	− 35.152**
	(15.905)	(15.277)	(17.359)
No direct link	− 131.704***	− 130.211***	− 137.716***
	(46.850)	(46.310)	(47.945)
Orig INstr	0.002***	0.002***	0.001***
	(0.001)	(0.000)	(0.000)
Orig OUTstr	0.003***	0.006***	
	(0.001)	(0.001)	
Orig INdeg	− 0.628***	− 0.782***	− 1.328***
	(0.134)	(0.154)	(0.238)
Orig OUTdeg	0.486***	0.219	0.939***
	(0.170)	(0.208)	(0.171)
Orig hub		− 551.749***	347.210***
		(174.576)	(52.380)
Orig betwenness		0.006***	− 0.004***
		(0.002)	(0.001)
Orig PGrank	− 2,436.400**		
	(1,026.057)		
Orig clustering	44,827.623	− 6,124.873	137,761.862***
	(38,193.997)	(38,562.569)	(45,560.299)
Dest INstr	0.015***	0.013***	0.011***
	(0.004)	(0.005)	(0.003)
Dest OUTstr	− 0.013	− 0.038	
	(0.010)	(0.024)	
Dest INdeg	2.419	1.886	1.818
	(1.522)	(1.326)	(1.329)
Dest OUTdeg	− 12.030***	− 11.322***	− 12.247***
	(2.884)	(2.716)	(2.860)
Dest hub		6,597.240*	50.692
		(3,997.418)	(408.636)
Dest betwenness		− 0.017	− 0.002
		(0.041)	(0.040)
Dest PGrank	− 10,935.922		
	(8,177.085)		
Dest clustering	− 203,987.148**	− 212,691.356*	− 195,663.331*
	(99,660.890)	(116,993.132)	(103,512.823)
Observations	88,752	88,752	88,752
$pseudoR^2$	0.154	0.154	0.153

The table reports the detailed results of the Tobit regressions discussed in "Regression analysis". Dummies for $\alpha \in (0, 0.5, 1)$ not shown. Clustering and PageRank measures have been multiplied by 1,000. Robust standard errors in parentheses. Significance level: *** $p<0.01$, ** $p<0.05$, * $p<0.1$.

#### Distance from origin of the shock

When the shock is domestic, a country will—everything else equal—bear a larger fraction of it and thus experience a larger caloric deficit.

On the other hand, when the shock originates abroad, the network distance we observe in our data ranges between 1 and 3, plus a number of country-pairs at infinite distance as there are no directed path going from one to the other. We have modeled distance by means of two indicator variables, one denoting an indirect connection between the origin of the shock and the one experiencing a demand deficit (that is, $distance(ORIG, DEST) \ge 2$ ), and the second taking value 1 if the distance is infinite. The baseline case is therefore when the country that receives the shock imports food from the country of where the shock originates. Network distance is negatively correlated with the magnitude of the demand deficit. In particular, not importing directly from the country originally hit by the shock is associated with an average reduction in the per capita caloric deficit of 25–27 kcals per day, while the distance from origin is infinite the deficit is substantially less severe (around − 130 kcals/person/day).

The relatively small effect of an indirect shock (coupled with a significance that does not reach the 5% level) indicates that in a highly connected food system shocks will not only affect direct partners, but reverberate globally.

#### Degree centrality

The magnitude of the final demand deficit correlates negatively with the out-degree of a country, which thus has more means to pass the shock on to other countries, while the positive correlation with the number of inward links is not significant. In quantitative terms, an additional export connection is associated (other things being equal) to a fall in the demand deficit of about 11–12 kcals/person/day.

A larger number of export partners for the country where the shock originates increases the magnitude of the final demand deficit, as a larger number of outward links spread the shock in multiple directions, while more inward links have a negative effect. In these cases, however, the size of the estimated coefficients is much smaller than in the case of destination countries.

#### Node strength

The number of import partners is a noisy measure of exposure to external shocks, because not all trade flows are equally important. When looking at the total magnitude of kcals that are either imported or exported by countries we observe that a larger amount of imports is associated with a larger demand deficit, and the same holds when a shock originates from a country that exports a lot. On the other hand, this feature does not play a significant role in shielding countries from the negative effects of a shock, although the estimated coefficient for the out-strength of destination countries takes a negative sign.

#### Import concentration

The concentration of imports, defined as the share of imports coming from the four most important trading partners (C4), has a negative effect on the demand deficit. This suggests that the relying on a small number of key suppliers shields countries from external shocks and that the usual diversification argument (whereby agents are better off when they source from multiple partners in order to edge against risk) seems not to apply here.

#### Local clustering coefficient

Countries featuring a high level of clustering experience, on average, lower demand deficits. On the other hand, the shock is not significantly correlated with the local clustering coefficient of the country of origin.

One way to rationalize this result is the fact that when a shock reaches a cluster, there are multiple channels that allow countries to dissipate it; moreover, it is less likely to disproportionately affect a single partner since withing the group of countries there will be strong interconnections.

#### Centrality

We experiment with different measures of network centrality, namely betweenness, hub score, and PageRank. We do not have a strong prior on the effect of centrality: while shocks originating in more central countries will probably affect a larger number of countries, the very fact that they travel further implies there is more room for it to dissipate and have a smaller effect on single countries. On the other hand, more central countries are more likely to be hit by external shocks, but should also have the ability to pass them on to more partners.

**Figure 5:**
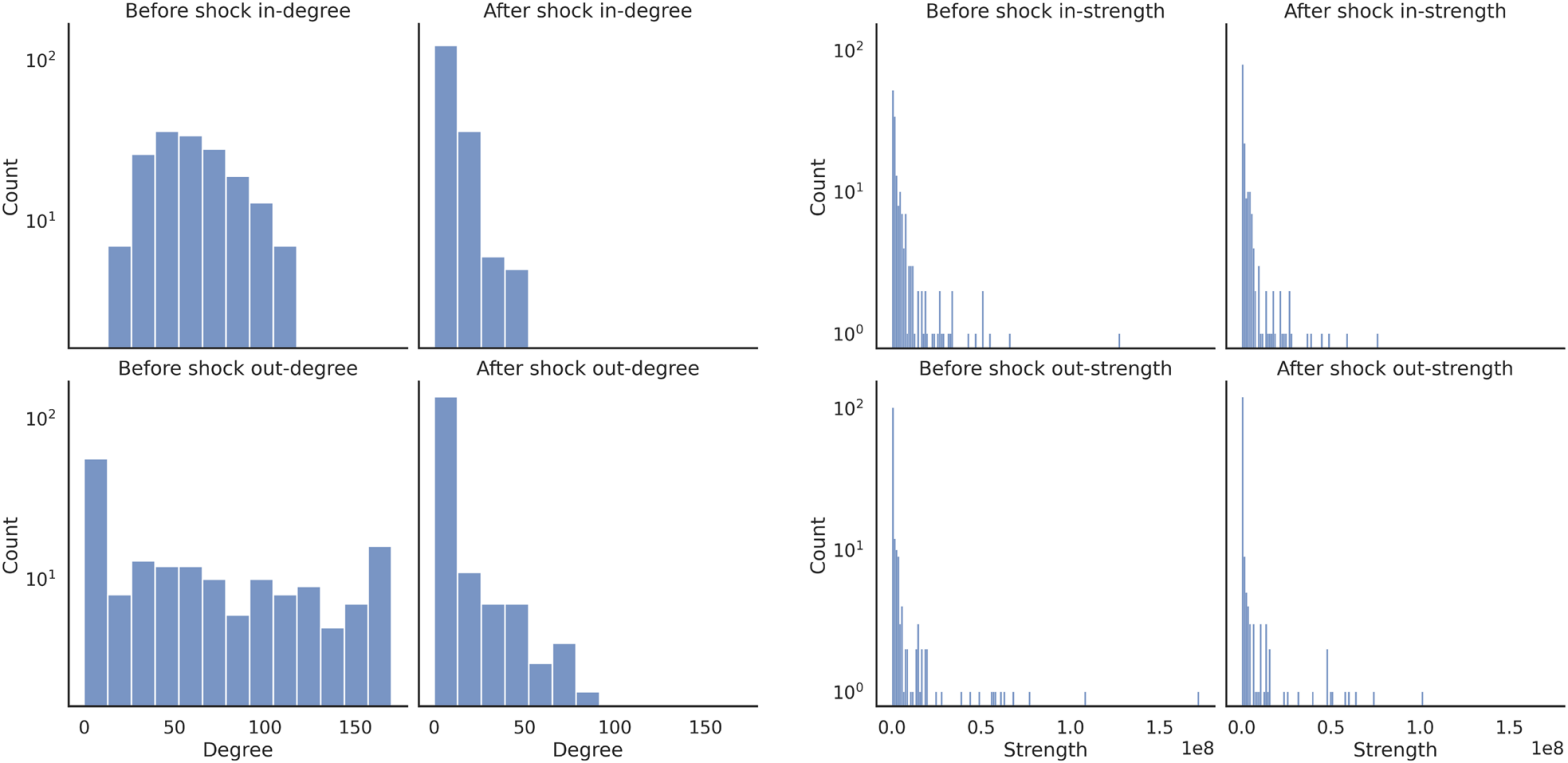
Diffusion of shock hitting the US: degree (left panel) and strength (right panel) distributions before and after the propagation.

When it comes to countries *receiving* an external shock, we find no significant correlation between any of the centrality measures and the size of the final demand deficit. On the contrary, the position of origin countries in the network does play a role. The hub score and, to a lesser extent, PageRank centrality are negatively correlated with the caloric deficit, whereas the correlation is positive when we look at betweenness centrality. Hubs, which export to several countries, thus appear to distribute the shock across multiple partners and this reduces the toll imposed on each one of them. On the other hand, high betweenness may signal the ability of a country to connect different parts of the network, but not necessarily through many links; as a result, when a shock originates in central country it may travel to distant countries yet generate, everything else equal, important consequences on trade partners (Fig. 5).
